# Unraveling the Link Between Fibromuscular Dysplasia and Acute Coronary Syndrome: A Comprehensive Case Report and Analysis

**DOI:** 10.7759/cureus.39605

**Published:** 2023-05-28

**Authors:** Mukosolu F Obi, Vikhyath Namireddy, Uzoma Ibebuogu, Shivani Dave, Hyun Joon Cho, Asim Elahi, Ariel Frederick

**Affiliations:** 1 Internal Medicine, Wyckoff Heights Medical Center, Brooklyn, USA; 2 Medicine, St. George's University School of Medicine, True Blue, GRD; 3 Cardiovascular Medicine, University of Tennessee Health Science Center, Memphis, USA; 4 Hospitalist, Saint Joseph London Hospital, London, USA; 5 Hospitalist, Pikeville Medical Center, Pikeville, USA

**Keywords:** genetic predisposition, left anterior descending artery (lad), acute chest syndrome (acs), renal fibromuscular dysplasia, coronary fibromuscular dysplasia

## Abstract

We present a rare case of fibromuscular dysplasia (FMD) manifesting in the mid to distal segment of the left anterior descending (LAD) artery, which led to the development of acute coronary syndrome (ACS) in our patient, highlighting the severe consequences of this vascular disorder. During the investigation of the patient's clinical symptoms, an unexpected incidental finding emerged, indicating bilateral FMD involvement of the renal arteries. This serendipitous discovery underscores the importance of comprehensive evaluation and thorough exploration when managing patients with FMD. We aim to shed light on the intriguing nature of FMD and emphasize the need for vigilant assessment to identify potential multi-vessel abnormalities, even beyond the primary affected site. We also aim to highlight the coronary artery manifestation of FMD as ACS and discuss its medical management.

## Introduction

Fibromuscular dysplasia (FMD) is a rare and intriguing vascular disorder that can have significant clinical implications. Its etiology remains elusive, and the exact cause of this condition is not yet fully understood [1]. The clinical manifestation of FMD depends on the arterial bed affected. The typical duration between the onset of the first symptom or sign and the diagnosis of FMD is four to nine years [1]. While there are no significant or definitive factors identified as the primary cause for the development of FMD, several studies have suggested multifactorial etiologies involving genetic, hormonal, and environmental factors [1].

Genetic predisposition appears to play a role in FMD, as familial cases have been reported in first-degree relatives of affected individuals, indicating a possible hereditary component. Studies have identified certain genetic variations associated with FMD, including mutations in genes involved in vascular development and extracellular matrix regulation with inheritance patterns of autosomal dominant with variable penetrance [1]. However, the genetic mechanisms underlying FMD are complex and require further investigation to elucidate their precise contribution. Hormonal influences, particularly estrogen, have also been implicated in the pathogenesis of FMD. The predominance of FMD in women, particularly during their reproductive years, suggests a potential hormonal influence on the development and progression of the disease, although FMD has not been associated with the use of oral contraceptives or the number of pregnancies [1]. Environmental factors such as smoking have also been linked to FMD development. Further studies and investigations are needed to understand the contributions of these factors to the development of FMD. In this report, we present an interesting case of FMD, specifically located in the mid to distal segment of the left anterior descending (LAD) artery, which resulted in the occurrence of acute coronary syndrome (ACS) in our patient. Coronary FMD is an emerging area of clinical research, as its imaging presentation does not involve the typical string of beads appearance but rather occurs in the distal coronary arteries.

## Case presentation

**Figure 1:**
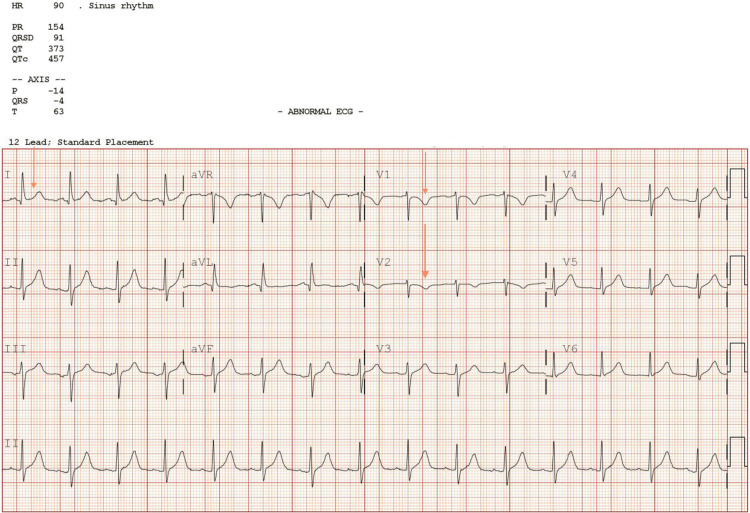
Electrocardiogram on admission: minimal ST elevation in lead I and T wave inversion in V1-V2

A 62-year-old female presented to the emergency department with sudden-onset chest pain radiating to the neck and left arm. The pain was severe, located in the center of the chest, rated as 10/10 in severity, and associated with vomiting. The patient reported that the episode had occurred suddenly in a car when returning from church. Additionally, she complained of intermittent bilateral back pain, which she had mentioned to her primary care provider and had been prescribed painkillers for. On arrival at the ED, her blood pressure was high, measuring 185/118 mmHg, with a heart rate of 95 beats per minute, and a respiratory rate of 18. An EKG was performed and it showed minimal ST elevation in lead I and T wave inversions in V1-V2 (Figure 1). Basic laboratory along with high-sensitivity troponin and urinalysis was done (Tables 1, 2). She denied any previous similar episodes and had not taken any medications to alleviate the pain but had instead come straight to the emergency department. During the physical examination, the patient was in moderate distress but alert and oriented to person, place, and time. Heart sounds S1 and S2 were present, with no murmurs, rubs, or gallops. There was no jugular vein distension, and her blood pressure was similar in both upper extremities. The patient's pain was not reproducible, and there was no bilateral lower extremity swelling. Lung auscultation revealed clear sounds bilaterally. The patient's medical history included hypertension, mixed hyperlipidemia, gastroesophageal reflux disease, breast argumentation, and a history of depression and anxiety. There was also a significant family history of cardiac disease, with the patient's brother having died of cardiac arrest at the age of 64 years. The differential diagnoses were ACS (non-ST-elevation myocardial infarction vs. unstable angina) and hypertensive emergency. Chest X-ray showed no focal infiltrates and perihilar interstitial prominence. Transthoracic echocardiography (TTE) was indicative of a left ventricular ejection fraction (LVEF) of 55-60%. There were no wall motion abnormalities or significant valvulopathy.

**Table 1:** Laboratory results: hematology showing WNL results NL: normal limit; WNL: within normal limits; WBC: white blood cells; MCV: mean corpuscular volume; MCH: mean corpuscular hemoglobin

Hematology	Results
WBC	6.39 x 1000/mm^3 ^(4.5–11 NL)
Hemoglobin	14.1 g/dL (12–15: adult female NL)
Hematocrit	41.8% (41–50 NL)
MCV	91.6 fL (80–100 NL)
MCH	30.9% (26–33 NL)
Platelet count	198 x 1000/mm^3 ^(150–450 NL)
International normalized ratio	1.00 (0.88–1.13 ratio)
Partial thromboplastin time	66.6 seconds (27.0–37.0)
Prothrombin time	11.8 seconds (10.2–13.3)

**Table 2:** Laboratory results: significant for elevated troponin, prediabetes, and hyperlipidemia NL: normal limit; GFR: glomerular filtration rate; LDL: low-density lipoproteins

Chemistry	Results
Urine toxicology	Negative
High-sensitive troponin – time 0	20.1 ng/mL (3.0–58.9 NL)
High-sensitive troponin – four hours later	1385.2 ng/mL (3.0–58.9 NL)
High-sensitive troponin – eight hours later	13784.4 ng/mL (3.0–58.9 NL)
Calcium	8.9 mg/dL (8.6–10.3 NL)
Sodium	142 mEq/L (136–145 NL)
Potassium	3.9 mEq/L (3.5–5.2 NL)
Chloride	106 mmol/L (96–106 NL)
CO_2_	27 mEq/L (23–29 NL)
Blood urea nitrogen	16 mg/dL (7–20 NL)
GFR	>60 (>60 NL)
Creatinine	0.81 mg/dL (0.7–1.3 NL)
Glucose	104 mmol/L (70–100 NL)
Triglycerides	90 mg/dL (2–150 NL)
LDL cholesterol	128 mg/dL (1–100 NL)
Total cholesterol	225 mg/dL (50–200 NL)
Magnesium	2.1 mg/dL (1.7–2.2 NL)
Phosphorus	3.4 mg/dL (3.4–4.5 NL)
Hemoglobin A1c	5.8% (4–5.6 NL)

Management

**Figure 2:**
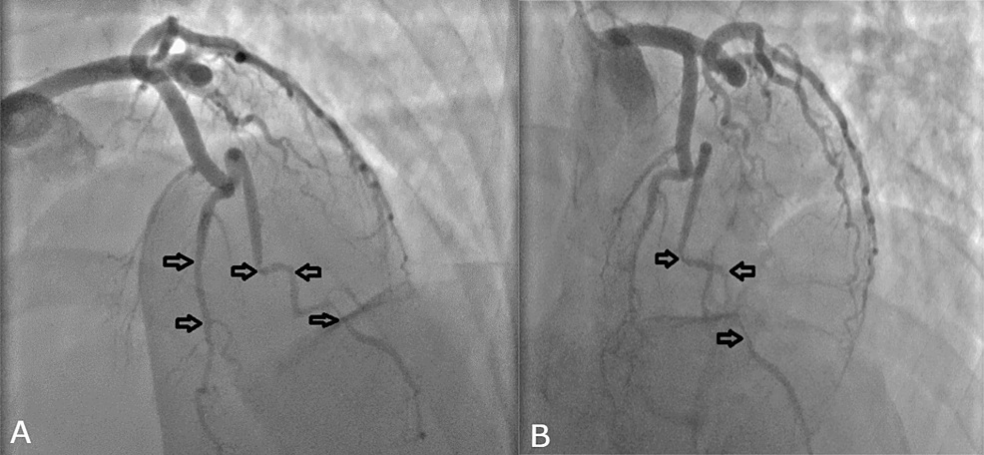
Left heart coronary artery catheterization findings A: Arrows indicate mild diffuse disease in the left circumflex artery, severe diffuse disease in the LAD artery, and stenosis in diagonal branch 1 (D1: culprit vessel). B. The image shows diffuse narrowing in distal LAD and D1 LAD: left anterior descending

**Figure 3:**
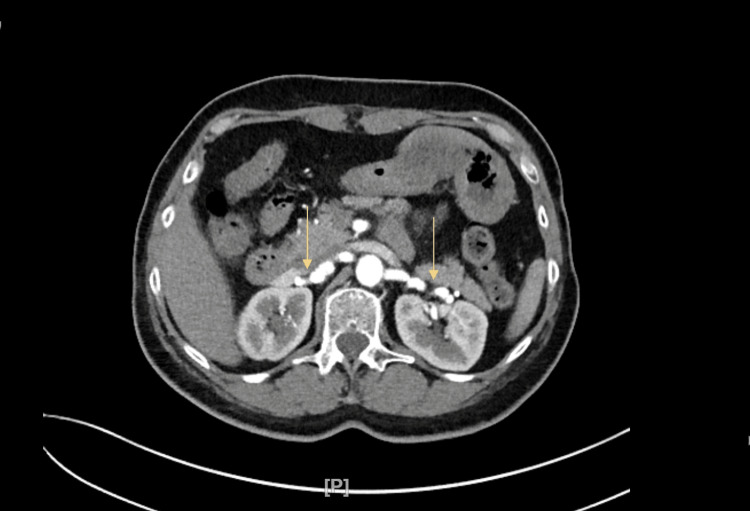
CT angiogram of the abdomen and pelvis with IV contrast The image shows the narrowing of the bilateral renal arteries, appearing as a string of beads CT: computed tomography

Upon arrival at the emergency department, the patient had high blood pressure, increased heart rate, and normal respiratory rate, level of consciousness, and stroke scale. She was immediately administered medication for chest pain, including sublingual nitroglycerin twice, 20 minutes apart, as well as aspirin and amlodipine. Her chest pain improved significantly after the medication was given. A point-of-care ultrasound (POCUS) was performed, which did not reveal any abnormalities in the aortic root, abdominal aorta, or pericardial effusion. However, the second troponin test returned significantly higher values than the first, indicating a cardiac event (Table 2). Repeat EKG was significant for T wave inversion in leads V4-V6. The patient was treated according to ACS protocol, including a heparin bolus, heparinization for 48 hours, and medication for hypertension. She was then taken to the cardiac catheterization laboratory, where significant results were observed (Figure 2, Table 3). CT angiogram (CTA) of the head/neck and CTA chest with IV contrast indicated no evidence of intracranial arterial thrombus, vascular malformation, or intracranial aneurysm. There was no evidence of dissection involving the visualized arterial vasculature, and no aneurysm or dissection. CTA of the abdomen and pelvis with IV contrast showed narrowing of the bilateral renal arteries, appearing as a string of beads (Figure 3). The patient was continued on heparin, aspirin, clopidogrel, and atorvastatin. Additionally, she was started on lisinopril to address her bilateral renal artery FMD and hypertension, with an expected ~30% increase in creatinine levels.

**Table 3:** Coronary angiography and abdominal aortogram results Coronary angiogram was significant for diffuse distal LAD disease with 90-95% stenosis. The abdominal aortogram was notable for right and left renal FMD LAD: left anterior descending; FMD: fibromuscular dysplasia

Coronary angiography	Abdominal aortogram
Dominance: right dominant. Right coronary artery: angiographically normal	Normal abdominal aorta
Left main: angiographically normal	Normal superior mesenteric/celiac artery
LAD artery	Normal inferior mesenteric
Distal LAD: diffusely diseased up to 90%; 95% stenosis, long segment disease, distal disease	Right renal artery (distal): FMD
Circumflex artery: mild luminal irregularities	Left renal artery (distal): FMD

Follow-up

The patient will remain on dual antiplatelet therapy (DAPT) for one year and undergo conservative management. An optimal medical therapy will be planned for the next six months. In addition to DAPT, the patient was discharged on amlodipine, lisinopril, high-intensity statin, and metoprolol titrate. If the patient continues to experience chest pain after the planned period of conservative management, balloon angiography will be considered.

## Discussion

FMD, also known as fibromuscular hyperplasia, is a rare nonatherosclerotic, noninflammatory vascular disease that mainly affects the small-to-medium-sized arteries in the body, such as the renal, extracranial carotid, and vertebral arteries. The clinical manifestations of FMD depend on the affected vessels. Renal artery involvement can cause hypertension, while carotid or vertebral artery involvement can lead to symptoms like dizziness, vertigo, tinnitus, transient ischemic attack (TIA), or stroke. Coronary artery involvement may cause ACS and myocardial ischemia. Due to the rarity of FMD and the nonspecific symptoms in terms of presentation, most cases are detected incidentally during imaging performed to evaluate other differential diagnoses.

There are three types of FMD, categorized based on the arterial layer involved. The intimal disease accounts for 1-2% of cases; medial FMD is the most common type and accounts for over 90% of cases, characterized by fibromuscular ridges that cause arterial stenosis with alternating smooth muscle degeneration, resulting in arterial dilation and producing the "string of beads" appearance on angiography. Periarterial FMD has a prevalence of 15-25% and is mainly found in female children, with a prevalence of less than 1% in adults [1].

The exact epidemiology of FMD remains unclear due to its rarity and the lack of standardized diagnostic criteria. However, it is estimated to affect approximately 3-4% of the population, with a higher prevalence among women than men, with a ratio of 9:1 [1]. FMD does not follow a simple inheritance pattern but rather appears to have a complex one. Studies show that the phenotypic expression of FMD varies among family members, indicating variable penetrance of vascular wall abnormalities in specific vascular beds [1,2]. Familial FMD tends to be more severe. Some patients with FMD have mutations in the transforming growth factor β1 (TGF-β1) and TGF-β2 genes, which are important for cell growth, differentiation, migration, and apoptosis. This suggests that these genes may play a role in the development of the disease, although further research is necessary to identify potential targets for treatment [1]. The pathogenesis of FMD is likely multifactorial and can result from a combination of genetic, environmental, and other factors. Damage and inflammation of the arterial wall caused by various factors may trigger abnormal growth and remodeling. FMD is more common in women, and it has been hypothesized that estrogen can stimulate the production of collagen and growth factors such as TGF-β, integrin-linked protein kinase 1 (ILK-1), and fibroblast growth factor (FGF), promoting smooth muscle proliferation and eventually leading to mechanical stress on arterial walls [2,3]. Treatment of FMD usually involves a combination of medications, lifestyle modifications, and, in some cases, interventional procedures such as angioplasty or stenting.

Diagnosing coronary artery involvement in FMD can be difficult, as the symptoms and imaging findings may be vague or may resemble other cardiac conditions. However, some patients with FMD experience myocardial infarction caused by coronary artery dissection, with the arterial lesions typically appearing in the mid to distal section of the LAD artery [3,4]. Physicians should consider the diagnosis of coronary FMD in any patient without cardiac risk factors but with ACS or new left ventricular dysfunction, presenting with isolated mid-distal coronary artery disease and normal coronary arteries elsewhere, particularly in middle-aged women [2]. The manifestations of noncoronary FMD may also aid in the diagnosis of coronary FMD. Diagnostic tests such as coronary angiography, intravascular ultrasound (IVUS), and cardiac MRI can be used to assess the extent and severity of coronary artery involvement in patients with suspected or confirmed FMD. Angiographic features of coronary FMD may include dissection, smooth narrowing or distal tapering, intramural hematoma, tortuosity, and spasm [2]. IVUS is a highly valuable imaging technique that can be used alongside other imaging methods to visualize the coronary or other vascular beds. When used in conjunction with angiography, IVUS can provide detailed information on the various layers of the arterial wall, which can aid in distinguishing between coronary artery manifestations of FMD and other types of coronary disease [2].

Managing coronary FMD poses significant challenges. The absence of specific treatment guidelines or evidence-based recommendations makes it difficult to establish a goal-directed therapy for this condition. Consequently, the management of coronary FMD relies heavily on case reports, limited case series, and extrapolation of therapies from non-randomized trials. In cases where patients with coronary FMD experience refractory ischemia despite receiving optimal medical treatment, the consideration for percutaneous coronary intervention or coronary artery bypass graft surgery arises. However, these invasive procedures are only recommended when the patient's ischemia remains unresponsive to medical therapy. It is worth noting that a conservative approach is generally preferred over invasive coronary intervention in FMD cases. This preference stems from the potential risk of sudden coronary artery dissection, which may heal naturally. If stenting is performed in the presence of a dissection flap that extends beyond the stented segment, it can lead to the formation of an intramural hematoma. Based on the available information, a reasonable treatment approach for patients with coronary FMD involves DAPT for one year, followed by aspirin therapy for an indefinite period. The use of beta-blockers is also considered, as they can reduce coronary shear forces that contribute to the risk of coronary dissection. It is important to emphasize that these treatment recommendations are derived from the current understanding of coronary FMD management, which is primarily based on observational data and clinical experience. Further research and evidence are necessary to develop more targeted and evidence-based therapies for coronary FMD [4].

## Conclusions

Coronary FMD remains a rare entity, often leading to challenges in recognition and diagnosis. However, it is crucial for physicians to be aware of this rare condition and its potential to manifest as ACS, particularly affecting the epicardial arteries, notably the mid-distal region of the LAD artery. The diagnostic journey for coronary FMD can be arduous, given the scarcity of evidence-based guidelines to guide treatment decisions. Therefore, each case necessitates an individualized approach, drawing upon the available knowledge and expertise to determine the most suitable therapeutic interventions. As physicians continue to unravel the intricacies of coronary FMD, further research and collaboration are warranted to enhance our understanding, improve diagnostic capabilities, and establish evidence-based treatment strategies for this intriguing vascular disorder.
